# Site Specific Mutation of the *Zic2* Locus by Microinjection of TALEN mRNA in Mouse CD1, C3H and C57BL/6J Oocytes

**DOI:** 10.1371/journal.pone.0060216

**Published:** 2013-03-28

**Authors:** Benjamin Davies, Graham Davies, Christopher Preece, Rathi Puliyadi, Dorota Szumska, Shoumo Bhattacharya

**Affiliations:** 1 Wellcome Trust Centre for Human Genetics, University of Oxford, Oxford, United Kingdom; 2 Department of Cardiovascular Medicine, University of Oxford, Oxford, United Kingdom; Montana State University, United States of America

## Abstract

Transcription Activator-Like Effector Nucleases (TALENs) consist of a nuclease domain fused to a DNA binding domain which is engineered to bind to any genomic sequence. These chimeric enzymes can be used to introduce a double strand break at a specific genomic site which then can become the substrate for error-prone non-homologous end joining (NHEJ), generating mutations at the site of cleavage. In this report we investigate the feasibility of achieving targeted mutagenesis by microinjection of TALEN mRNA within the mouse oocyte. We achieved high rates of mutagenesis of the mouse *Zic2* gene in all backgrounds examined including outbred CD1 and inbred C3H and C57BL/6J. Founder mutant *Zic2* mice (eight independent alleles, with frameshift and deletion mutations) were created in C3H and C57BL/6J backgrounds. These mice transmitted the mutant alleles to the progeny with 100% efficiency, allowing the creation of inbred lines. Mutant mice display a curly tail phenotype consistent with *Zic2* loss-of-function. The efficiency of site-specific germline mutation in the mouse confirm TALEN mediated mutagenesis in the oocyte to be a viable alternative to conventional gene targeting in embryonic stem cells where simple loss-of-function alleles are required. This technology enables allelic series of mutations to be generated quickly and efficiently in diverse genetic backgrounds and will be a valuable approach to rapidly create mutations in mice already bearing one or more mutant alleles at other genetic loci without the need for lengthy backcrossing.

## Introduction

The ability to precisely modify the mouse genome experimentally has had a considerable impact over the last 25 years in diverse areas of biomedical research and has made the mouse one of the most important model organisms in the laboratory today. Alterations in the genome are conventionally made by the process of gene targeting in ES cells [1]. Using this method, whole genes or exons can be deleted from the mouse genome and the phenotypic consequences of these knock-out models can deliver important information concerning gene function. With the advent of genome sequencing and more recently genetic association studies implicating genes as risk factors for disease susceptibility, a bottleneck in functional analysis is emerging [2], exacerbated by discoveries concerning the importance of non-coding RNA [3]. Internationally funded consortia aimed at knocking-out all protein coding genes [4] and knock-outs projects addressing microRNA [5] are beginning to tackle this bottleneck. These initiatives have facilitated a wider access to mutant mouse technology within the research community.

As an alternative approach, new technologies for targeted mutagenesis based on sequence specific nucleases are emerging [6], [7]. These enzymes are dimers of hybrid proteins consisting of a DNA binding domain coupled to a nuclease domain, frequently Fok1. The monomers are engineered to bind to specific sequences on opposing strands of DNA in between which the Fok1 dimer introduces a double strand break (DSB). Cellular mechanisms act at the DSB and repair the break, frequently by a process known as Non-Homologous End Joining (NHEJ). This DSB repair mechanism can be mutagenic with the deletion or insertion of a few base pairs occurring at the site of strand breakage [8]. The introduction of DSBs can thus be used to introduce mutations at specific sequences.

Two classes of nucleases are available for targeted mutagenesis which differ in the type of DNA binding domain. Zinc Finger Nucleases (ZFNs) use a zinc finger DNA binding module which can be engineered to specific sequences [9]. Modules of individual fingers recognizing 3 base-pair DNA sequences can be combined to create sequence specific DNA binding domains [10]. It has become clear, however, that simple modular assembly can be unreliable as frequently the specificities of the zinc finger-DNA interactions depend on the context of neighbouring fingers and the DNA sequence [11]. Consequently more elaborate randomized pool screening methodologies are recommended for the selection of a zinc finger array with reliable DNA binding properties [12].

The second class of nucleases, the TALENs utilize the DNA binding domain of a family of transcriptional regulators from the plant pathogen *Xanthomonas*, which act to modulate expression of plant bacterial defence genes to facilitate infection [13]. These domains are characterised by a series of almost identical repeat modules which differ only at two amino acid residues. These two residues define the nucleotide base to which each so called repeat-variable di-residue domain (RVD) preferentially binds [14], [15] and consequently, sequence specific DNA binding domains can be constructed by assembling multiple RVDs in the required order [16].

Both classes of enzymes have been shown to be active in mammalian cells where they can introduce a single DSB within the genome at the address specified by the design of the DNA binding site [17], [18]. Zinc finger nucleases have been applied successfully within the fertilized embryo of almost all commonly used model organism, including the mouse [19]. The widespread use of these enzymes as an alternative to conventional gene targeting in ES cells, however, has been limited and one reason for this might be the difficulties in establishing the context dependent zinc finger selection strategies within the laboratory.

In contrast, the simplicity and reliability of a single protein module contacting a specific nucleotide for the TALE domain makes the TALENs more amenable for widespread use in the research community. To date TALENs have been transfected into mammalian stem cells [20], [21] and have also been introduced directly as mRNA into the fertilized oocyte to achieve site specific mutation in rat [22], pig [23], zebrafish [24]–[26], Xenopus [27] and Drosophila [28]. In this study, we have addressed for the first time the feasibility of a TALEN mediated mutagenesis approach in the mouse and show site specific mutation of the *Zic2* gene at high efficiency in multiple genetic backgrounds by microinjection of TALEN mRNA into the oocyte.

*Zic2* belongs to a family of zinc finger transcription factors which represent the vertebrate homologues of the *Drosophila* pair rule gene *odd-paired* [29]. In mouse, *Zic2* is encoded by three exons, with the DNA binding C2H2-type zinc finger motifs being encoded by the latter part of exon1 and the entirety of exon 2. In vertebrates *Zic2* is widely expressed in the developing nervous system [30] and studies with mutant mice have demonstrated a role for this gene in the temporal regulation of neurulation [31]. Consistently in humans, clinical data has revealed that mutations in human *ZIC2* account for a large number of cases of the neural tube closure defect, holoprosencephaly, one of the most common congenital abnormalities in humans [32], [33].

We have chosen *Zic2* for this proof-of-concept study firstly, because existing targeted models have only resulted in reduced *Zic2* expression [31], secondly, because heterozygous *Zic2* loss-of-function results in a visible phenotype, albeit with variable penetrance [34] and thirdly, as the TALEN mutagenesis approach has the potential to generate an allelic series of mutations which, when targeted to the functional zinc finger domain of this transcription factor, could model many of the human mutations associated with holoprosencephaly [33].

## Materials and Methods

### TALEN construction

Plasmids encoding TALEN enzymes were constructed by Golden Gate assembly of the required RVDs into pTAL3 using the Golden Gate TALEN and TAL Effector Kit [35] (Addgene #1000000016). TALEN-A was designed against the sequence 5′-ATCTCTGCAAGATGT-3′ for the sense strand using the RVD array NI-NG-HD-NG-HD-NG-NN-HD-NI-NI-NN-NI-NG-NN-NG and 5′-GCTTCCGCAACGAGCT-3′ on the antisense strand using the RVD array NN-HD-NG-NG-HD-HD-NN-HD-NI-NI-HD-NN-NI-NN-HD-NG. TALEN-B was designed against the sequence 5′- GTCCACACCTCAGAT-3′ for the sense strand using the RVD array NN-NG-HD-HD-NI-HD-NI-HD-HD-NG-HD-NI-NN-NI-NG and 5′- AGGACTTGTCACACAT-3′ on the antisense strand using the RVD array NI-NN-NN-NI-HD-NG-NG-NN-NG-HD-NI-HD-NI-HD-NI-NG. The coding regions of each of the plasmids were cloned into the mammalian expression vector, pcDNA3 (Life Technologies) via AflII and XhoI to generate plasmids pcDNA3-TALEN-A-Fwd, pcDNA3-TALEN-A-Rev encoding the sense and antisense components of TALEN-A, and pcDNA3-TALEN-B-Fwd and pcDNA-TALEN-B-Rev, encoding the sense and antisense components of TALEN-B.

### Validation of TALEN enzymes

Two oligonucleotides harbouring both the TALEN-A and TALEN-B binding sites were annealed together to create an adaptor with EcoRI and BamHI overhangs (TALEN-A: 5′-AATTATCTCTGCAAGATGTGTGTCAAGTCCTACACGCATCCCAGCTCGTTGCGGAAGC-3′; 5′- GATCGCTTCCGCAACGAGCTGGGATGCGTGTAGGACTTGACACACATCTTGCAGAGAT-3′; TALEN-B: 5′- AATTTGTCCACACCTCAGATAAGCCCTATCTCTGCAAGATGTGTGACAAGTCCTT-3; 5′- GATCAAGGACTTGTCACACATCTTGCAGAGATAGGGCTTATCTGAGGTGTGGACA-3′) which was cloned into pRGS (Toolgen) [36] to create reporter plasmids, pRGS-Zic2A and pRGS-Zic2B, containing a upstream dsRed expression cassette separated from an out-of-frame eGFP cassette by the Zic2 TALEN-A and TALEN-B target sequences respectively. The relevant reporter vector was transfected into HEK293T (ATCC CRL-11268) cells together with combinations of the TALEN-A and TALEN-B expression plasmid using Fugene HD Transfection Reagent (Promega) following the manufacturer's recommendation. Cells were cultured for 48 hours and assessed for red and green fluorescence.

### Preparation and microinjection of TALEN mRNA

mRNA was generated with T7 RNA polymerase from 1 ug of linearized pcDNA3-TALEN construct using the mMessage mMachine T7 Kit (Life Technologies), according to the manufacturer's instructions. The resulting mRNAs were purified using the MEGAclear kit (Life Technologies), following the manufacturer's instructions exactly. mRNAs were eluted in 2×50 ul of the kit's Elution Buffer, prewarmed. Purified mRNA was diluted to 5 ng/ul in 1 mM Tris.HCl pH 7.5/0.1 mM EDTA and was microinjected into the cytoplasm of fertilized oocytes, prepared from superovulated plugged females at 0.5 dpc. Injected oocytes were cultured overnight in KSOM microdrops and the resulting two cell embryos were either left in culture for 2–3 days until the blastocyst stage or were immediately transferred surgically to pseudopregnant CD1 foster mothers at 0.5 dpc.

### Mutation detection and genotyping

Blastocysts or ear biopsies from pups were digested with proteinase K in lysis buffer (10 mM Tris-HCl pH 8.0, 50 mM KCl, 0.45% NP40, 0.45% TWEEN 20), heat inactivated and used directly in a PCR reaction to amplify the *Zic2* exon 2 using primers Zic2-F (5′-GGAGAAACCTTTCCAGTGTG-3′) and Zic2-R (5′-GAAGACAAAAGCCGGGAGTG-3′). Approximately 400 ng of the amplification product was used in Cel1 nuclease assay (Surveyor MDK kit, Transgenomic), according to the manufacturer's instructions. Cel1 digested products were analysed by gel electrophoresis.

The amplification product from putative mutants was directly sequenced using Sanger sequencing or individual amplicons were cloned by TA cloning (pGEM-T System, Promega) and sequenced from the resulting plasmids. Mutations were elucidated by aligning individual sequence reads or were extrapolated from mixed sequencing reads using CodonCode Aligner 3.7 (Codoncode corporation).

### Mice

C57BL/6J and CD-1 mice were sourced from Charles River Laboratories. C3H/HeH mice were sourced from MRC Harwell. Mice were housed in individually ventilated cages and all husbandry and procedures occurred under Home Office Project License approval.

## Results

**Figure 1:**
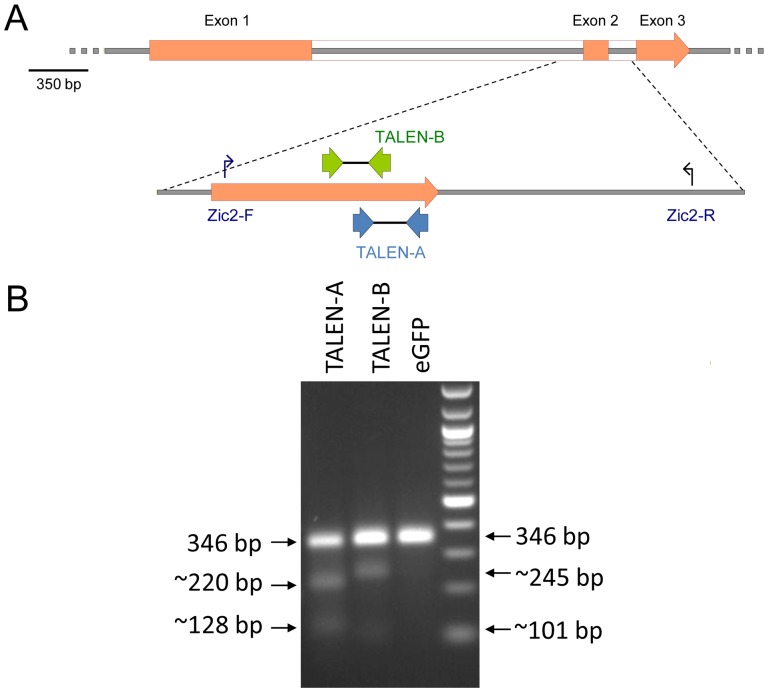
*Zic2* genomic structure and TALEN binding sites. A) Genomic structure of the murine *Zic2* (upper panel) with an enlargement of exon 2 (lower panel), showing the binding sites of the two monomers for TALEN-A and TALEN-B together with the binding sites of the PCR primers, Zic2-F and Zic2-R used to genotype the mutant alleles. B) Example of the Cel1 endonuclease assay showing cleavage of the PCR amplicon from example heterozygous mutant CD1 embryos, injected with TALEN-A mRNAs, TALEN-B mRNAs and control eGFP mRNA. The fragments obtained correspond to the predicted cleavage of the 346 bp amplicon within the spacer region of the TALEN-A and TALEN-B recognition sites as expected.

Two independent TALEN pairs (TALEN-A and TALEN-B) were designed to *Zic2* exon 2 (ENSMUSE00000551739) using the TAL Effector Nucleotide Targeter 2.0 [37] and were constructed by Golden Gate cloning of RVD modules into the PthXo1 TAL effector-Fok1 nuclease scaffold [35] (Figure 1a). Each individual TALEN construct was cloned into a mammalian expression vector and the two pairs were functionally validated using a NHEJ reporter assay [36] in Hek293T cells (Figure S1). The second exon of *Zic2* was chosen for TALEN induced mutagenesis as it encodes two of the critical C2H2-type zinc finger domains and thus represents a significant portion of the putative DNA binding domain. Mutations in this region have been associated with holoprosencephaly in humans and are thus hypothesized to disrupt *Zic2* function [33].

**Figure 2:**
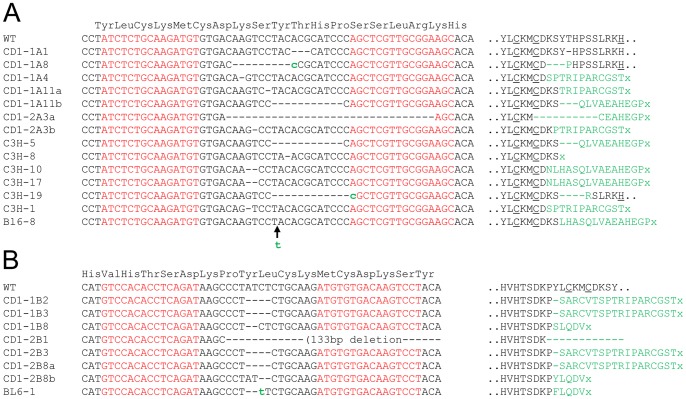
Sequence information of *Zic2* mutant alleles. A) Sequences obtained from the mutant blastocysts (CD1) or from the founder lines of mutant mice (C3H and C57BL/6J) generated following microinjection of TALEN-A mRNAs. B) Sequences obtained from the mutant blastocysts (CD1) or from the founder line of mutant mice (C57BL/6J) generated following microinjection of TALEN-B mRNAs. The DNA sequences to which the TALEN monomers were designed are highlighted in red. Nucleotide mutations and insertions (shown with arrows) are shown in lower case and are highlighted in green. The reading frame of the ZIC2 protein is shown above the wild-type sequences and the predicted consequences of the mutation on the amino acid sequences are shown to the right of the DNA sequences. Divergence (missense/deletion etc) from the wild-type peptide sequence is shown in green and premature Stop codons are shown as x. Critical cysteine and histidine residues of the 5^th^ zinc finger domain are underlined.

To assess their ability to introduce mutations specifically within the *Zic2* gene in vivo, mRNA was generated for each of the TALEN monomers, and was microinjected in pairs into the cytoplasm of fertilized outbred CD-1 oocytes. Following injection, oocytes were cultured for 3 days in vitro until the blastocyst stage. The resulting blastocysts were lysed and a region of genomic DNA encompassing *Zic2* exon 2 was amplified by PCR using primers Zic2-F and Zic2-R and screened for the presence of TALEN induced mutations by the Cel1 endonuclease assay (Figure 1b). Microinjection of TALEN-A mRNAs resulted in a mutation rate of 46% and microinjection of TALEN-B mRNAs resulted in a mutation rate of 40% (Table 1). PCR products were either directly sequenced or cloned and sequenced to establish the nature of the TALEN induced mutations (Figure 2). The sequence analysis revealed that the majority of mutated embryos were heterozygous for a *Zic2* mutation. Three embryos however revealed the presence of 3 independent sequence traces (wild-type and two different mutations), implying that these embryos were mosaics of 2 different mutations, presumably as a result of TALEN induced mutation after the first cleavage event. One blastocyst, CD1-1B1, was found to be heterozygous for a large complex deletion; the complete alignment of this mutant sequence with the wild-type sequence is shown in Figure S2.

**Table 1:** Microinjection Results.

Mouse strain	TALEN-A		TALEN-B	
	No. of pups or blastocysts	No of mutants recovered	No. of pups or blastocysts	No of mutants recovered
CD1	15	7 (46%)	15	6 (40%)
C3H/HeH	26	6 (23%)	-	-
C57BL/6J	10	1 (10%)	10	1 (10%)

Having shown that microinjection of TALEN mRNA can be used to achieve site specific mutation in the fertilized oocyte, injected embryos were investigated for their ability to develop to term and thus produce lines of *Zic2* mutant mice. Fertilized oocytes from two commonly used inbred mouse strains, C3H/HeH and C57BL/6J, were microinjected with either TALEN-A (C3H/HeH and C57BL/6J) or TALEN-B mRNAs (C57BL/6J), cultured overnight to the two cell stage and transferred into pseudopregnant females. Resulting pups were analysed for mutation at the *Zic2* gene using the Cel1 endonuclease assay and DNA sequencing as previously described. Table 1 summarizes the results of the microinjection and the mutations generated are shown in Figure 2, along with the putative amino-acid sequence that would result from translation of the mutant alleles. In total six independent founder lines harbouring *Zic2* mutations were generated on a C3H/HeH background and two founders lines were generated on a C57BL/6J background.

**Figure 3:**
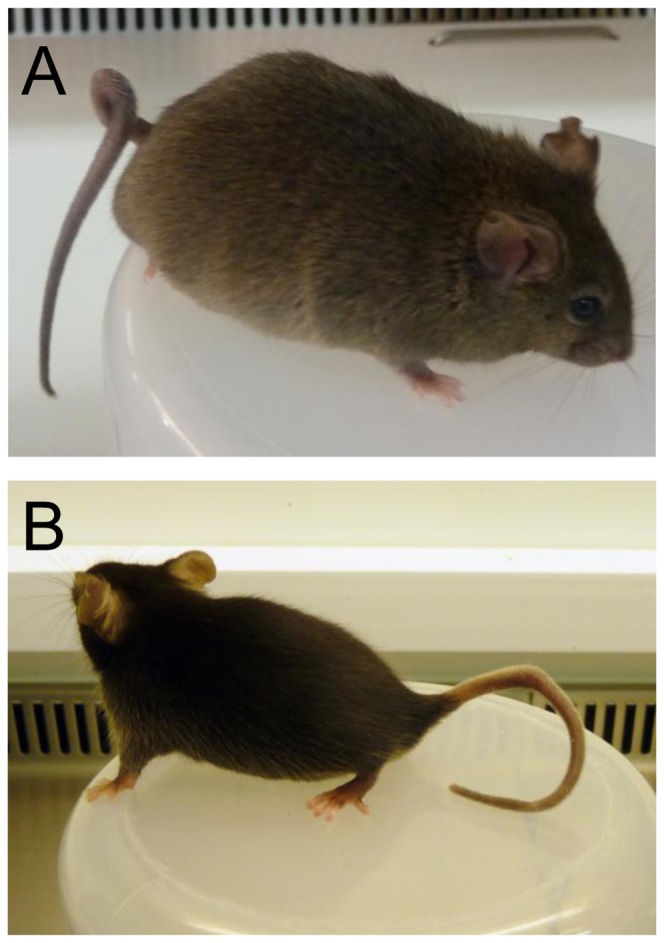
Examples of the curly tail phenotype seen in some of the mutant founder mice. A) TALEN-A C3H founder 10. B) TALEN-B C57BL/6 founder 1.

Loss of function of *Zic2* is associated with a curly tail phenotype of variable penetrance in heterozygous mice, indicative of deficits in neural tube closure [34]. Even before the molecular analysis was performed, it was clear that a number of the pups generated following TALEN mRNA microinjection showed a curly tail phenotype (Figure 3). The pups displaying this phenotype were found to be heterozygous for a *Zic2* mutation (founder C3H-10, C3H-19, generated with TALEN-A, and founder BL6-1, generated with TALEN-B).

5 independent founder mice generated on the C3H/HeH background were mated with wild-type C3H/HeH mice to assess whether the de novo *Zic2* mutations could be transmitted through the germ layer. All founder mice tested exhibited normal fertility and were found to transmit the mutations at the expected Mendelian ratios without any statistically significant deviation from the expected distribution (Table 2). For all of these 5 lines (including the lines for which the founder mouse showed no visible tail phenotype), the curly tail phenotype appeared sporadically in a subset of the heterozygous mice, consistent with the known variable penetrance of this loss-of-function phenotype [34].

**Table 2:** Mutant *Zic2* Founder line characterization.

			F1 Genotypes	
Line	Mutation	Phenotype	WT	Het
C3H-5	p.(Tyr401Gln fs*12)	Curly tail in F1	15	12
C3H-8	p.(Tyr401*)	Curly tail in F1	13	18
C3H-10	p.(Lys399Asn fs*17)	Curly tail in F0 and F1	18	11
C3H-17	p.(Lys399Asn fs*17)	Curly tail in F1	7	9
C3H-19	p.(Tyr401_Ser405del insArg)	Curly tail in F0 and F1	12	9

Overall, the mutation rates of 25% on a C3H/HeH background and mutation rates of 10% on a C57BL/6J background, confirm the TALEN mRNA microinjection method to be an efficient and practical method of targeted mutagenesis in the mouse.

## Discussion

In this study we have used TALENs to achieve site specific sequence mutation of the mouse genome directly within the fertilized oocyte and have shown that the method is feasible in several commonly used inbred and outbred strains of mice. Mutant alleles were transmitted according to Mendelian ratios and phenotypes consistent with loss of *Zic2* function were displayed in some of the heterozygous mice. As has already been shown in a variety of other model organisms (reviewed in [38]), the results of this study suggest that TALEN mutagenesis technology has the potential to dramatically impact mutant mouse production within the research community.

The majority of the mutant alleles generated in this study are predicted to seriously disrupt the 5^th^ zinc finger of the *Zic2* transcription factor. All but three of the mutant alleles generated contain frameshift mutations which lead to the loss of the critical histidine or cysteine residues that comprise the classic C2H2-type zinc finger structure and are required for the coordination of the zinc ion within the DNA binding motif. Furthermore, the frameshift mutations also lead to a premature termination of the protein, meaning that the entirety of exon 3, encoding 117 amino acid residues of unknown function would be absent from the translated product. Those alleles which retain the critical residues and do not cause a frame-shift and subsequent premature stop codon (CD1-1A1, CD1-1A8 and C3H-19), have altered spacing between the cysteine cluster and the histidine cluster and are thus expected to have a disrupted domain with a compromised ability to bind to DNA. Accordingly, all of the alleles analysed as lines of mutant mice (including line C3H-19 with its in-frame deletion) display the variable penetrant curly tail phenotype, entirely consistent with *Zic2* heterozygous loss-of-function [34].

The mouse is already a well characterised model organism which is permissive for genome engineering of considerable complexity and precision, thanks to the availability and ease of manipulation of pluripotent embryonic stem (ES) cells. The targeted ablation of specific sequences within the mouse genome has traditionally been achieved by the process of homologous recombination in these cells. Recombinant ES cells are screened for the required homologous recombination event and are subsequently injected into pre-implantation embryos, where the stem cells are required to contribute to the development of the germ cells, allowing the mutant strain of mice to be established. This technique demands elaborate and extensive molecular biology for the construction of the targeting vector and the screening of recombinant ES cells. Furthermore, the production of the mutant strain can necessitate lengthy breeding procedures, as frequently the desirable ES cell clones may have been compromised in their ability to contribute to the germ line. Conservative estimation of production times for loss-of-function Knock-outs using this technique are between 8 and 12 months until chimeras of breeding age are generated.

In contrast, using the TALEN mediated mutagenesis approach described in this study, the molecular biology required for the construction of the TALENs and the screening of mutations is a great deal simpler than the techniques required for targeting vector construction and screening for homologous recombination events. This is partly due to the reliability and simplicity of the modular construction kit and the free availability of the resources and kits within the research community. The production time for site specific mutant mice can be reduced to 4 months for the generation of breeding age founders (assuming 1 month for the assembly and validation of the TALENs and 3 months for the microinjection and raising of litters). The approach thus has the potential to vastly reduce the development time for loss of function mouse models.

An additional advantage for the TALEN approach is that theoretically it can be achieved on complex genetic backgrounds. Mutations can be introduced directly into oocytes derived from strains of mice which already harbour multiple transgenes and mutant alleles. The ability to simply add mutations to an existing background directly obviates the need to interbreed models and could save many generations of interbreeding or backcrossing.

A potential disadvantage of the TALEN approach is that the nature of the mutation is uncontrolled and mutations which preserve the reading frame can be generated. However, the high rates of mutagenesis allow for the generation of multiple founders, allowing non-disruptive mutations to be discarded. Indeed the production of multiple mutant founder lines all harbouring different mutations can be informative as an allelic series of mutations for a specific gene can easily be generated. Using the data from this study as an example, of the 8 live *Zic2* mutant alleles characterised, only one allele had an in-frame deletion and the remaining 7 led to frameshift and downstream nonsense mutations.

A further disadvantage may lie in off-target cleavage and subsequent mutagenesis. Preliminary studies using candidate off-target loci screening have revealed the levels of TALEN off-target mutagenesis to be low but detectable [20], [39]. However, comparable with ENU mutagenesis, the husbandry of the mutant strains necessitates the breeding to wild-type mice and thus it is expected that unselected off-target mutations, if not genetically linked, would simply be lost. It is becoming clear that there are always limitations and potential sources of unexplored genomic variation and mutational “noise” with every technology. For example, screening of ES cells by comparative genomic hybridization techniques have revealed ES cell karyotypes to be far from stable in culture with frequently copy number variations arising in cell culture [40]. Offsite mutations via the TALEN approach could thus be considered to be comparable to this as yet unexplored source of experimental noise.

An interesting observation in this study was the presence of multiple mutations in certain mutant embryos – three mutants genotyped at the blastocyst stage clearly revealed complex mixes of mutations. This observation is very similar to the mosaicism which has been reported for zinc finger nuclease mediated mutagenesis in the mouse [19]. The observation of mosaicism suggests that the nucleases responsible for the sequence specific DSB persist and are active within the embryo after the first few cleavage events, subsequently there is a risk that microinjected embryos result in mutant mosaics. Interestingly, the genotyping and the germline transmission analysis of the live mutant founders provide no evidence for mosaicism. The reason for this discrepancy is unclear, but it should always be assumed that there is a risk of mosaicism and multiple mutations with this method and thus the F1 generation must be screened carefully.

Homozygous or compound heterozygous mutants were not detected in this study. Complete loss of *Zic2* function is not compatible with development, which might explain this observation, as complete loss of function mice would not have been recovered. However, analysis of mutants at the blastocyst stage of development also failed to reveal mutation of both copies of the *Zic2* gene. Recent reports in zebrafish using new TALEN protein scaffolds and nuclear domains harbouring hyperactive mutations suggest that mutation of both autosomal copies of target genes can be achieved using the TALEN mutagenesis approach [24], [41]. Although a systematic comparison of the efficiency of TALENs built with the different scaffolds is not yet available, it is likely that the scaffolds used for TALEN construction within this study are of more modest activity which might explain the lack of homozygous loss of function alleles detected.

Overall the results of this study suggest that TALEN mRNA microinjection may provide a fast and efficient approach for the production of simple loss-of-function mouse models. The availability and constant improvement of fast and efficient open-source methods and resources for the construction of TALENs makes this method a viable alternative to conventional gene targeting in embryonic stem cells for the production of loss-of-function alleles. A recent report of DBS induced homology directed repair within the mouse oocytes following microinjection of zinc finger nucleases with an oligo donor [42], may widen the application of this important new technology for genome engineering in the mouse.

## Supporting Information

Figure S1 **In vitro testing of TALEN activity.** A) Principle of the NHEJ reporter assay to demonstrate functionality of the TALENs in vitro. A reporter construct expresses dsRed under the control of a constitutive CMV promoter, but the downstream eGFP cistron is initially out-of-frame and is not expressed. Upon cleavage of the intervening sequence by a functional TALEN, the error prone NHEJ repair leads to insertion or deletion of nucleotides at the cleavage site, reconstituting the eGFP reading frame. B). Fluorescent photomicrographs of reporter and TALEN expression plasmid transfected HEK293T cells. AF, AR, BF, BR signifies the addition of combinations of pcDNA3-TALEN-A-Fwd, pcDNA3-TALEN-A-Rev, pcDNA3-TALEN-B-Fwd and pcDNA3-TALEN-B-Rev respectively. Left hand panel shows the dsRed fluorescence of the reporter plasmid and right hand panels show the eGFP fluorescence of the reconstituted eGFP reading frame. Significant eGFP fluorescence was seen only in transfection combinations receiving both components of the TALEN pair.
(TIF)

Figure S2 **Alignment of the sequence of mutant blastocyst CD1-1B1 with wild-type** ***Zic2*** **.**
(TIF)
