# Supplementary figures and images for: Site Specific Mutation of the Zic2 Locus by Microinjection of TALEN mRNA in Mouse CD1, C3H and C57BL/6J Oocytes
Source: PLoS One. 2013 Mar 28;8(3):e60216. doi: 10.1371/journal.pone.0060216 (PMC3610929; doi:10.1371/journal.pone.0060216)

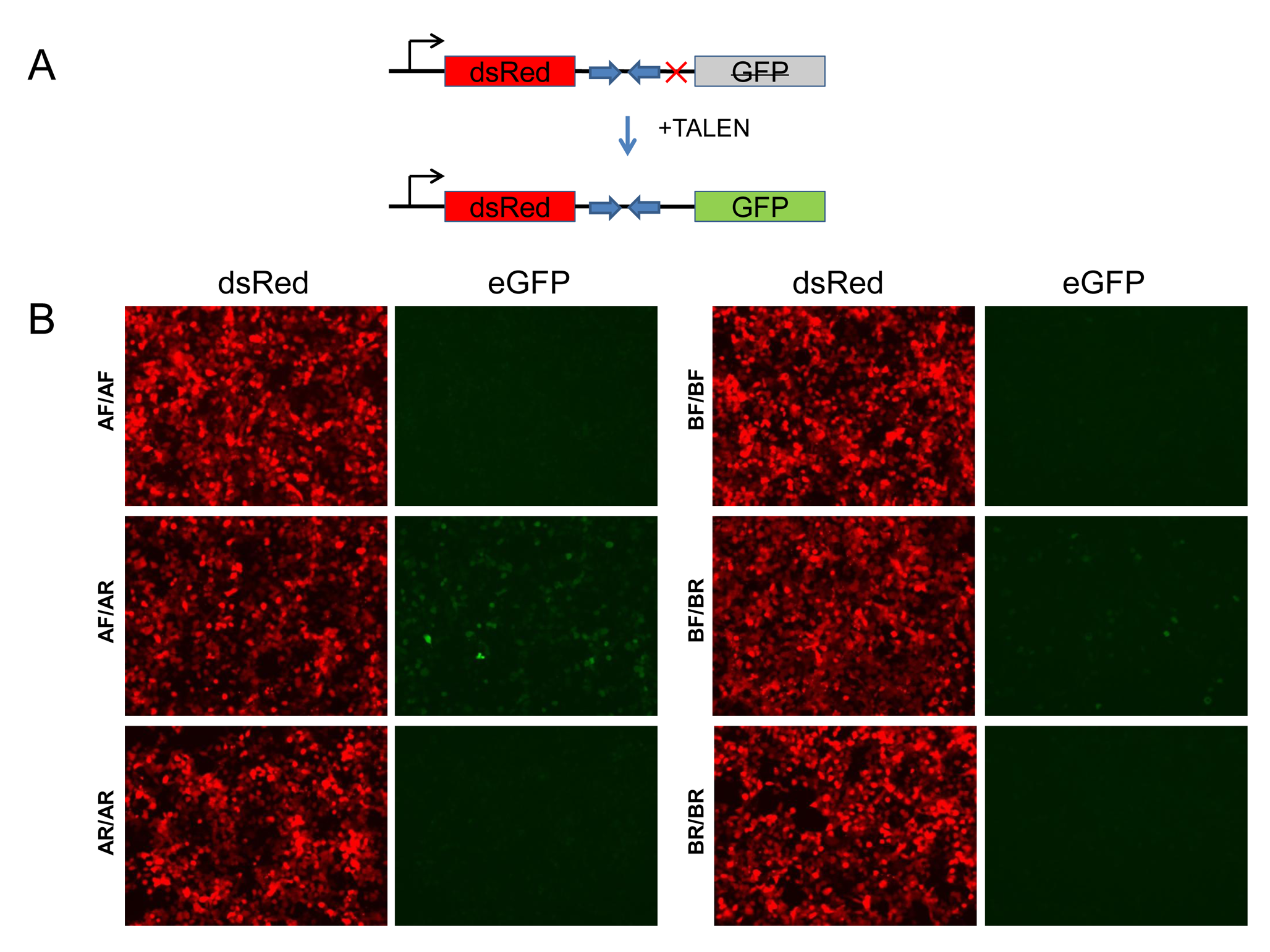

Supplement: Figure S1 — In vitro testing of TALEN activity. A) Principle of the NHEJ reporter assay to demonstrate functionality of the TALENs in vitro. A reporter construct expresses dsRed under the control of a constitutive CMV promoter, but the downstream eGFP cistron is initially out-of-frame and is not expressed. Upon cleavage of the intervening sequence by a functional TALEN, the error prone NHEJ repair leads to insertion or deletion of nucleotides at the cleavage site, reconstituting the eGFP reading frame. B). Fluorescent photomicrographs of reporter and TALEN expression plasmid transfected HEK293T cells. AF, AR, BF, BR signifies the addition of combinations of pcDNA3-TALEN-A-Fwd, pcDNA3-TALEN-A-Rev, pcDNA3-TALEN-B-Fwd and pcDNA3-TALEN-B-Rev respectively. Left hand panel shows the dsRed fluorescence of the reporter plasmid and right hand panels show the eGFP fluorescence of the reconstituted eGFP reading frame. Significant eGFP fluorescence was seen only in transfection combinations receiving both components of the TALEN pair. (TIF) [file pone.0060216.s001.tif]

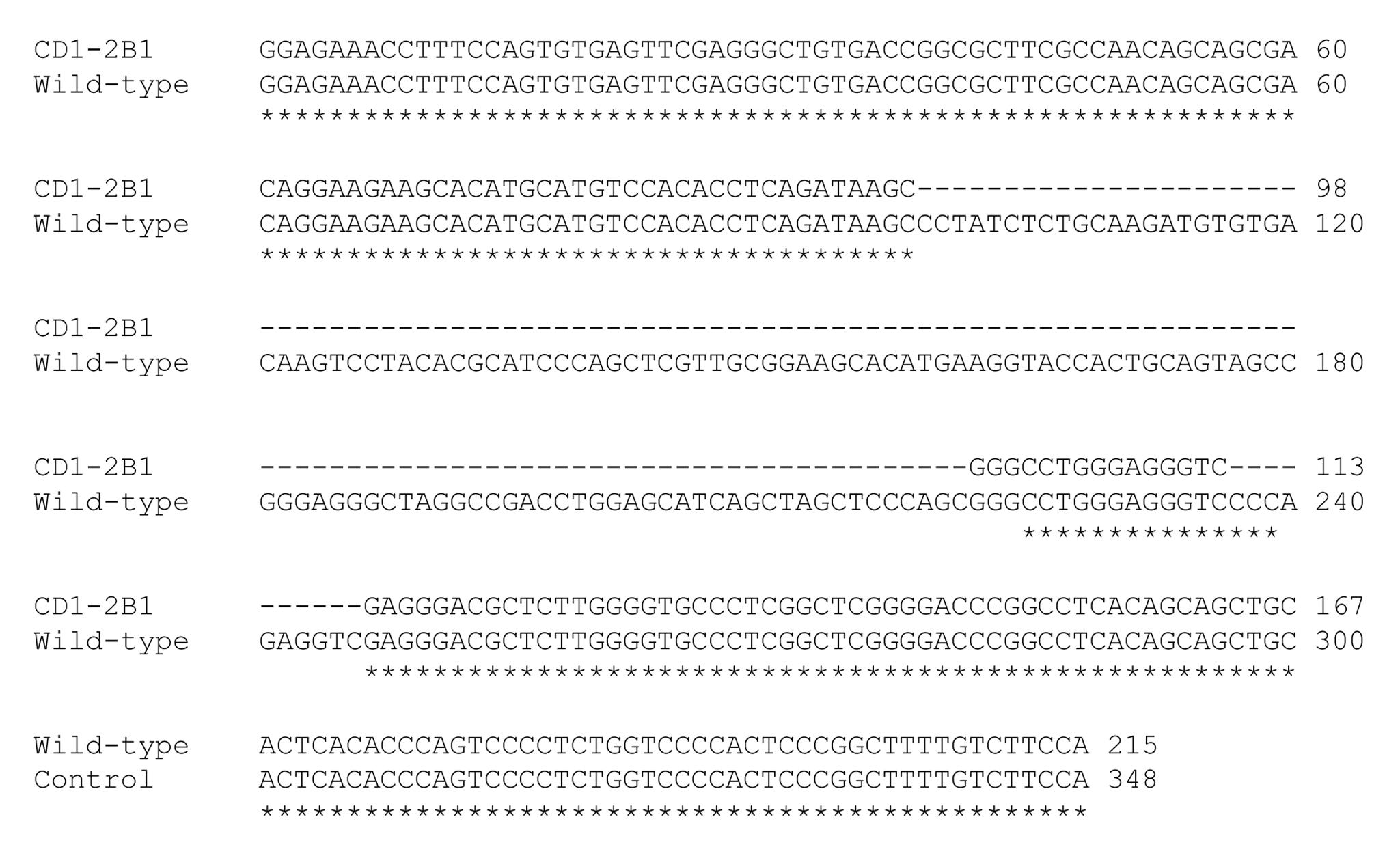

Supplement: Figure S2 — Alignment of the sequence of mutant blastocyst CD1-1B1 with wild-type Zic2 . (TIF) [file pone.0060216.s002.tif]
